# Metaheuristic Algorithms for Convolution Neural Network

**DOI:** 10.1155/2016/1537325

**Published:** 2016-06-08

**Authors:** L. M. Rasdi Rere, Mohamad Ivan Fanany, Aniati Murni Arymurthy

**Affiliations:** ^1^Machine Learning and Computer Vision Laboratory, Faculty of Computer Science, Universitas Indonesia, Depok 16424, Indonesia; ^2^Computer System Laboratory, STMIK Jakarta STI&K, Jakarta 12140, Indonesia

## Abstract

A typical modern optimization technique is usually either heuristic or metaheuristic. This technique has managed to solve some optimization problems in the research area of science, engineering, and industry. However, implementation strategy of metaheuristic for accuracy improvement on convolution neural networks (CNN), a famous deep learning method, is still rarely investigated. Deep learning relates to a type of machine learning technique, where its aim is to move closer to the goal of artificial intelligence of creating a machine that could successfully perform any intellectual tasks that can be carried out by a human. In this paper, we propose the implementation strategy of three popular metaheuristic approaches, that is, simulated annealing, differential evolution, and harmony search, to optimize CNN. The performances of these metaheuristic methods in optimizing CNN on classifying MNIST and CIFAR dataset were evaluated and compared. Furthermore, the proposed methods are also compared with the original CNN. Although the proposed methods show an increase in the computation time, their accuracy has also been improved (up to 7.14 percent).

## 1. Introduction

Deep learning (DL) is mainly motivated by the research of artificial intelligent, in which the general goal is to imitate the ability of human brain to observe, analyze, learn, and make a decision, especially for complex problem [1]. This technique is in the intersection amongst the research area of signal processing, neural network, graphical modeling, optimization, and pattern recognition. The current reputation of DL is implicitly due to drastically improve the abilities of chip processing, significantly decrease the cost of computing hardware, and advanced research in machine learning and signal processing [2].

In general, the model of DL technique can be classified into discriminative, generative, and hybrid models [2]. Discriminative models, for instance, are CNN, deep neural network, and recurrent neural network. Some examples of generative models are deep belief networks (DBN), restricted Boltzmann machine, regularized autoencoders, and deep Boltzmann machines. On the other hand, hybrid model refers to the deep architecture using the combination of a discriminative and generative model. An example of this model is DBN to pretrain deep CNN, which can improve the performance of deep CNN over random initialization. Among all of the hybrid DL techniques, metaheuristic optimization for training a CNN is the focus of this paper.

Although the sound character of DL has to solve a variety of learning tasks, training is difficult [3–5]. Some examples of successful methods for training DL are stochastic gradient descent, conjugate gradient, Hessian-free optimization, and Krylov subspace descent.

Stochastic gradient descent is easy to implement and also fast in the process for a case with many training samples. However, this method needs several manual tuning scheme to make its parameters optimal, and also its process is principally sequential; as a result, it was difficult to parallelize them with graphics processing unit (GPU). Conjugate gradient, on the other hand, is easier to check for convergence as well as more stable to train. Nevertheless, CG is slow, so that it needs multiple CPUs and availability of a vast number of RAMs [6].

Hessian-free optimization has been applied to train deep autoencoders [7], proficient in handling underfitting problem, and more efficient than pretraining + fine tuning proposed by Hinton and Salakhutdinov [8]. On the other side, Krylov subspace descent is more robust and simpler than Hessian-free optimization as well as looks like it is better for the classification performance and optimization speed. However, Krylov subspace descent needs more memory than Hessian-free optimization [9].

In fact, techniques of modern optimization are heuristic or metaheuristic. These optimization techniques have been applied to solve any optimization problems in the research area of science, engineering, and even industry [10]. However, research on metaheuristic to optimize DL method is rarely conducted. One work is the combination of genetic algorithm (GA) and CNN, proposed by You and Pu [11]. Their model selects the CNN characteristic by the process of recombination and mutation on GA, in which the model of CNN exists as individual in the algorithm of GA. Besides, in recombination process, only the layers weights and threshold value of C1 (convolution in the first layer) and C3 (convolution in the third layer) are changed in CNN model. Another work is fine-tuning CNN using harmony search (HS) by Rosa et al. [12].

In this paper, we compared the performance of three metaheuristic algorithms, that is, simulated annealing (SA), differential evolution (DE), and HS, for optimizing CNN. The strategy employed is looking for the best value of the fitness function on the last layer using metaheuristic algorithm; then the results will be used again to calculate the weights and biases in the previous layer. In the case of testing the performance of the proposed methods, we use MNIST (Mixed National Institute of Standards and Technology) dataset. This dataset comprises images of digital handwritten digits, containing 60,000 training data items and 10,000 testing data items. All of the images have been centered and standardized with the size of 28 × 28 pixels. Each pixel of the image is represented by 0 for black and 255 for white, and in between are different shades of gray [13].

This paper is organized as follows: Section 1 is an introduction, Section 2 explains the used metaheuristic algorithms, Section 3 describes the convolution neural networks, Section 4 gives a description of the proposed methods, Section 5 presents the result of simulation, and Section 6 is the conclusion.

## 2. Metaheuristic Algorithms

Metaheuristic is well known as an efficient method for hard optimization problems, that is, the problems that cannot be solved optimally using deterministic approach within a reasonable time limit. Metaheuristic methods work for three main purposes: for fast solving of problem, for solving large problems, and for making a more robust algorithm. These methods are also simple to design as well as flexible and easy to implement [14].

In general, metaheuristic algorithms use the combination of rules and randomization to duplicate the phenomena of nature. The biological system imitations of metaheuristic algorithm, for instance, are evolution strategy, GA, and DE. Phenomena of ethology for examples are particle swarm optimization (PSO), bee colony optimization (BCO), bacterial foraging optimization algorithms (BFOA), and ant colony optimization (ACO). Phenomena of physics are SA, microcanonical annealing, and threshold accepting method [15]. Another form of metaheuristic is inspired by music phenomena, such as HS algorithm [16].

Classification of metaheuristic algorithm can also be divided into single-solution-based and population-based metaheuristic algorithm. Some of the examples for single-solution-based metaheuristic are the noising method, tabu search, SA, TA, and guided local search. In the case of metaheuristic based on population, it can be classified into swarm intelligent and evolutionary computation. The general term of swarm intelligent is inspired by the collective behavior of social insect colonies or animal societies. Examples of these algorithms are GP, GA, ES, and DE. On the other side, the algorithm for evolutionary computation takes inspiration from the principles of Darwinian for developing adaptation into their environment. Some examples of these algorithms are PSO, BCO, ACO, and BFOA [15]. Among all these metaheuristic algorithms, SA, DE, and HS are used in this paper.

### 2.1. Simulated Annealing Algorithm

SA is a technique of random search for the problem of global optimization. It mimics the process of annealing in material processing [10]. This technique was firstly proposed in 1983 by Kirkpatrick et al. [17].

The principle idea of SA is using random search, which not only allows changes that improve the fitness function but also maintains some changes that are not ideal. As an example, in minimum optimization problem, any better changes that decrease the fitness function value *f*(*x*) will be accepted, but some changes that increase *f*(*x*) will also be accepted with a transition probability (*p*) as follows:

(1) $$\begin{array}{c} p=\exp\left(\;\frac{-\Delta E}{kT}\;\right), \end{array}$$

where Δ*E* is the energy level changes, *k* is Boltzmann's constant, and *T* is temperature for controlling the process of annealing. This equation is based on the Boltzmann distribution in physics [10]. The following is standard procedure of SA for optimization problems:

(1) *Generate the solution vector*: the initial solution vector is randomly selected, and then the fitness function is calculated.
(2) *Initialize the temperature*: if the temperature value is too high, it will take a long time to reach convergence, whereas a too small value can cause the system to miss the global optimum.
(3) *Select a new solution*: a new solution is randomly selected from the neighborhood of the current solution.
(4) *Evaluate a new solution*: a new solution is accepted as a new current solution depending on its fitness function.
(5) *Decrease the temperature*: during the search process, the temperature is periodically decreased.
(6) *Stop or repeat*: the computation is stopped when the termination criterion is satisfied. Otherwise, steps (2) and (6) are repeated.

### 2.2. Differential Evolution Algorithm

Differential evolution was firstly proposed by Price and Storn in 1995 to solve the Chebyshev polynomial problem [15]. This algorithm is created based on individuals difference, exploiting random search in the space of solution, and finally operates the procedure of mutation, crossover, and selection to obtain the suitable individual in system [18].

There are some types in DE, including the classical form DE/rand/1/bin; it indicates that in the process of mutation the target vector is randomly selected, and only a single different vector is applied. The acronym of bin shows that crossover process is organized by a rule of binomial decision. The procedure of DE algorithm is shown by the following steps:

(1) * Determining parameter setting*: population size is the number of individuals. Mutation factor (F) controls the magnification of the two individual differences to avoid search stagnation. Crossover rate (CR) decides how many consecutive genes of the mutated vector are copied to the offspring.
(2) *Initialization of population*: the population is produced by randomly generating the vectors in the suitable search range.
(3) *Evaluation of individual*: each individual is evaluated by calculating their objective function.
(4) * Mutation operation*: mutation adds identical variable to one or more vector parameters. In this operation, three auxiliary parents (*x*_*M*_^*p*1^, *x*_*M*_^*p*2^, *x*_*M*_^*p*3^) are selected randomly, in which they will participate in mutation operation to create a mutated individual *x*_*M*_^mut^ as follows:
  (2) $$\begin{array}{c} x_{M}^{mut}=x_{M}^{p1}+F\left(\;x_{M}^{p2}-x_{M}^{p3}\;\right), \end{array}$$
 where *p*1, *p*2, *p*3 ∈ {1,2,…, *N*} and *N* = *p*1 ≠ *p*2 ≠ *p*3.
(5) * Combination operation*: recombination (crossover) is applied after mutation operation.
(6) * Selection operation*: this operation determines whether the offspring in the next generation should become a member of the population or not.
(7) * Stopping criterion*: the current generation is substituted by the new generation until the criterion of termination is satisfied.

### 2.3. Harmony Search Algorithm

Harmony search algorithm is proposed by Geem et al. in 2001 [19]. This algorithm is inspired by the musical process of searching for a perfect state of harmony. Like harmony in music, solution vector of optimization and improvisation from the musician are analogous to structures of local and global search in optimization techniques.

In improvisation of the music, the players sound any pitch in the possible range together that can create one vector of harmony. In the case of pitches creating a real harmony, this experience is stored in the memory of each player and they have the opportunity to create better harmony next time [16]. There are three possible alternatives when one pitch is improvised by a musician: any one pitch is played from her/his memory, a nearby pitch is played from her/his memory, and an entirely random pitch is played with the range of possible sound. If these options are used for optimization, they have three equivalent components: the use of harmony memory, pitch adjusting, and randomization. In HS algorithm, these rules are correlated with two relevant parameters, that is, harmony consideration rate (HMCR) and pitch adjusting rate (PAR). The procedure of HS algorithm can be summarized into five steps as follows [16]:

1. *Initialize the problem and parameters*: in this algorithm, the problem can be maximum or minimum optimization, and the relevant parameters are HMCR, PAR, size of harmony memory, and termination criterion.
2. *Initialize harmony memory*: the harmony memory (HM) is usually initialized as a matrix that is created randomly as a vector of solution and arranged based on the objective function.
3. *Improve a new harmony*: a vector of new harmony is produced from HM based on HMCR, PAR, and randomization. Selection of new value is based on HMCR parameter by range 0 through 1. The vector of new harmony is observed to decide whether it should be pitch-adjusted using PAR parameter. The process of pitch adjusting is executed only after a value is selected from HM.
4. *Update harmony memory*: the new harmony substitutes the worst harmony in terms of the value of the fitness function, in which the fitness function of new harmony is better than worst harmony.
5. *Repeat (3) and (4) until satisfying the termination criterion*: in the case of meeting the termination criterion, the computation is ended. Alternatively, process (3) and (4) are reiterated. In the end, the vector of the best HM is nominated and is reflected as the best solution for the problem.

## 3. Convolution Neural Network

CNN is a variant of the standard multilayer perceptron (MLP). A substantial advantage of this method, especially for pattern recognition compared with conventional approaches, is due to its capability in reducing the dimension of data, extracting the feature sequentially, and classifying one structure of network [20]. The basic architecture model of CNN was inspired in 1962, from visual cortex proposed by Hubel and Wiesel.

In 1980, Fukushimas Neocognitron created the first computation of this model, and then in 1989, following the idea of Fukushima, LeCun et al. found the state-of-the-art performance on a number of tasks for pattern recognition using error gradient method [21].

**Figure 1:**
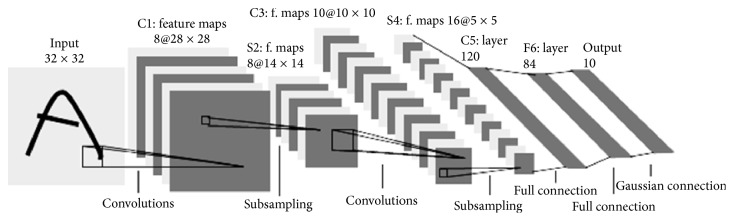
Architecture of CNN by LeCun et al. (LeNet-5).

The classical CNN by LeCun et al. is an extension of traditional MLP based on three ideas: local receptive fields, weights sharing, and spatial/temporal subsampling. These ideas can be organized into two types of layers, which are convolution layers and subsampling layers. As is showed in Figure 1, the processing layers contain three convolution layers C1, C3, and C5, combined in between with two subsampling layers S2 and S4 and output layer F6. These convolution and subsampling layers are structured into planes called features maps.

In convolution layer, each neuron is linked locally to a small input region (local receptive field) in the preceding layer. All neurons with similar feature maps obtain data from different input regions until the whole of plane input is skimmed, but the same of weights is shared (weights sharing).

In subsampling layer, the feature maps are spatially downsampled, in which the size of the map is reduced by a factor 2. As an example, the feature map in layer C3 of size 10 × 10 is subsampled to a conforming feature map of size 5 × 5 in the subsequent layer S4. The last layer is F6 that is the process of classification [21].

Principally, a convolution layer is correlated with some feature maps, the size of the kernel, and connections to the previous layer. Each feature map is the results of a sum of convolution from the maps of the previous layer, by their corresponding kernel and a linear filter. Adding a bias term and applying it to a nonlinear function, the *k*th feature map *M*_*ij*_^*k*^ with the weights *W* ^*k*^ and bias *b*_*k*_ is obtained using the tanh function as follows:

(3) $$\begin{array}{c} M_{ij}^{k}=\tanh\left(\;{\left(\;W^{k}\times x\;\right)}_{ij}+b_{k}\;\right). \end{array}$$

The purpose of a subsampling layer is to reach spatial invariance by reducing the resolution of feature maps, in which each pooled feature map relates to one feature map of the preceding layer. The subsampling function, where *a*_*i*_^*n*×*n*^ is the inputs, *β* is a trainable scalar, and *b* is trainable bias, is given by the following equation:

(4) $$\begin{array}{c} a_{j}=\tanh\left(\;\beta \underset{N\times N}{\sum }a_{i}^{n\times n}+b\;\right). \end{array}$$

After several convolutions and subsampling, the last structure is classification layer. This layer works as an input for a series of fully connected layers that will execute the classification task. It has one output neuron every class label, and in the case of MNIST dataset, this layer contains ten neurons corresponding to their classes.

## 4. Design of Proposed Methods

The architecture of this proposed method refers to a simple CNN structure (LeNet-5), not a complex structure like AlexNet [22]. We use two variations of design structure. First is i-6c-2s-12c-2s, where the number of C1 is 6 and that of C3 is 12. Second is i-8c-2s-16c-2s, where the number of C1 is 8 and that of C3 is 18. The kernel size of all convolution layers is 5 × 5, and the scale of subsampling is 2. This architecture is designed for recognizing handwritten digits from MNIST dataset.

In this proposed method, SA, DE, and HS algorithm are used to train CNN (CNNSA, CNNDE, and CNNHS) to find the condition of best accuracy and also to minimize estimated error and indicator of network complexity. This objective can be realized by computing the lost function of vector solution or the standard error on the training set. The following is the lost function used in this paper:

(5) $$\begin{array}{c} y=\frac{1}{2}{\left(\;\frac{\sum_{i=N}^{N}{\left(o-u\right)}^{2}}{N}\;\right)}^{0.5}, \end{array}$$

where *o* is the expected output, *u* is the real output, and *N* is some training samples. In the case of termination criterion, two situations are used in this method. The first is when the maximum iteration has been reached and the second is when the loss function is less than a certain constant. Both conditions mean that the most optimal state has been achieved.

### 4.1. Design of CNNSA Method

Principally, algorithm on CNN computes the values of weight and bias, in which on the last layer they are used to calculate the lost function. These values of weight and bias in the last layer are used as solution vector, denoted as *x*, to be optimized in SA algorithm, by adding Δ*x* randomly.

Δ*x* is the essential aspect of this proposed method. Selection in the proper of this value will significantly increase the accuracy. For example, in CNNSA to one epoch, if Δ*x* = 0.0008 × rand, then the accuracy is 88.12, in which this value is 5.73 greater than the original CNN (82.39). However, if Δ*x* = 0.0001 × rand, its accuracy is 85.79 and its value is only 3.40 greater than the original CNN.

Furthermore, this solution vector is updated based on SA algorithm. When the termination criterion is satisfied, all of weights and biases are updated for all layers in the system.  Algorithm 1 is the CNNSA algorithm of the proposed method.

### 4.2. Design of CNNDE Method

At the first time, this method computes all the values of weight and bias. The values of weight and bias on the last layer (*x*) are used to calculate the lost function, and then by adding Δ*x* randomly, these new values are used to initialize the individuals in the population.

Similar to CNNSA method, selection in the proper of Δ*x* will significantly increase the value of accuracy. In the case of one epoch in CNNDE as an example, if Δ*x* = 0.0008 × rand, then the accuracy is 86.30, in which this value is 3.91 greater than the original CNN (82.39). However, if Δ*x* = 0.00001 × rand, its accuracy is 85.51.

Furthermore, these individuals in the population are updated based on the DE algorithm. When the termination criterion is satisfied, all of weights and biases are updated for all layers in the system.  Algorithm 2 is the CNNDE algorithm of the proposed method.

### 4.3. Design of CNNHS Method

At the first time like CNNSA and CNNDE, this method computes all the values of weight and bias. The values of weight and bias on the last layer (*x*) are used to calculate the lost function, and then by adding Δ*x* randomly, these new values are used to initialize the harmony memory.

In this method, Δ*x* is also an important aspect, while selection of the proper of Δ*x* will significantly increase the value of accuracy. For example of one epoch in CNNHS (i-8c-2s-16c-2s), if Δ*x* = 0.0008 × rand, then the accuracy is 87.23, in which this value is 7.14 greater than the original CNN (80.09). However, if Δ*x* = 0.00001 × rand, its accuracy is 80.23; the value is only 0.14 greater than CNN.

Furthermore, this harmony memory is updated based on the HS algorithm. When the termination criterion is satisfied, all of weights and biases are updated for all layers in the system.  Algorithm 3 is the CNNHS algorithm of the proposed method.

## 5. Simulation and Results

**Figure 2:**
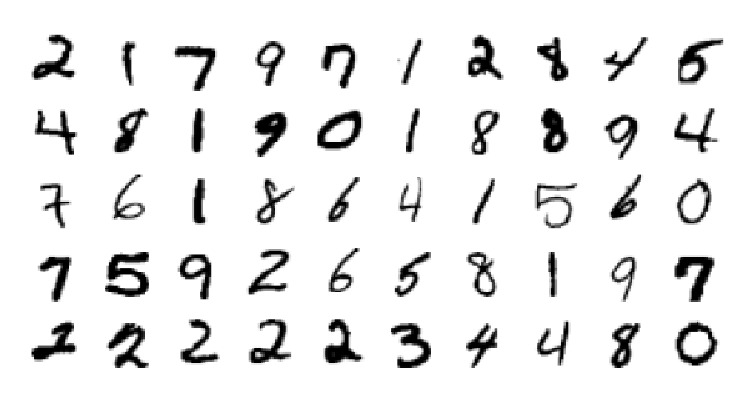
Example of some images from MNIST dataset.

In this paper, the primary goal is to improve the accuracy of original CNN by using SA, DE, and HS algorithm. This can be performed by minimizing the classification task error tested on the MNIST dataset. Some of the example images for MNIST dataset are shown in Figure 2.

In CNNSA experiment, the size of neighborhood was set = 10 and maximum of iteration (maxit) = 10. In CNNDE, the population size = 10 and maxit = 10. In CNNHS, the harmony memory size = 10 and maxit = 10. Since it is difficult to make sure of the control of parameter, in all of the experiment the values of *c* = 0.5 for SA, F = 0.8 and CR = 0.3 for DE, and HMCR = 0.8 and PAR = 0.3 for HS. We also set the parameter of CNN, that is, the learning rate (*α* = 1) and the batch size (100).

As for the epoch parameter, the number of epochs is 1 to 10 for every experiment. All of the experiment was implemented in MATLAB-R2011a, on a personal computer with processor Intel Core i7-4500u, 8 GB RAM running memory, Windows 10, with five separate runtimes. The original program of this simulation is DeepLearn Toolbox from Palm [23].

**Figure 3:**
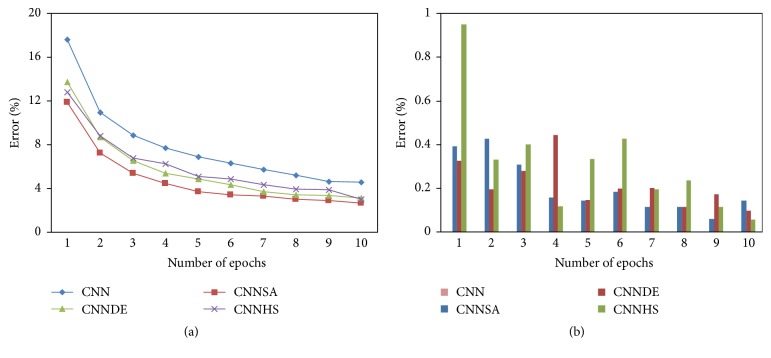
Error and its standard deviation (i-6c-2s-12c-2s).

**Figure 4:**
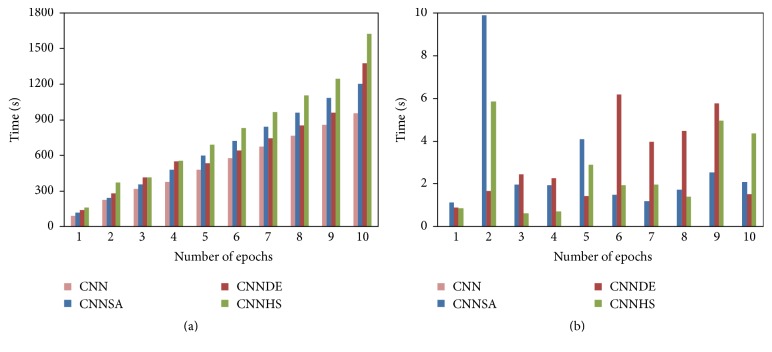
Computation time and its standard deviation (i-6c-2s-12c-2s).

**Figure 5:**
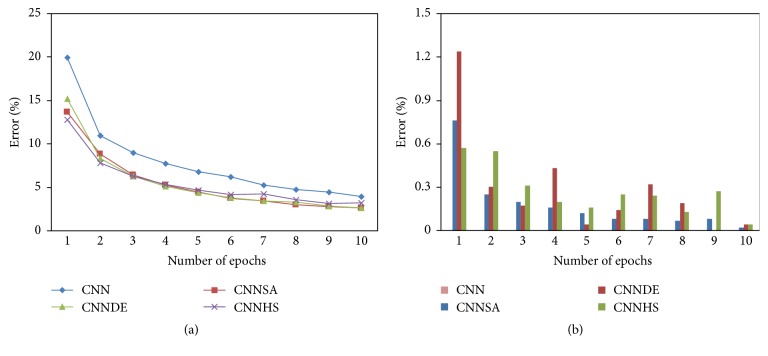
Error and its standard deviation (i-8c-2s-16c-2s).

**Figure 6:**
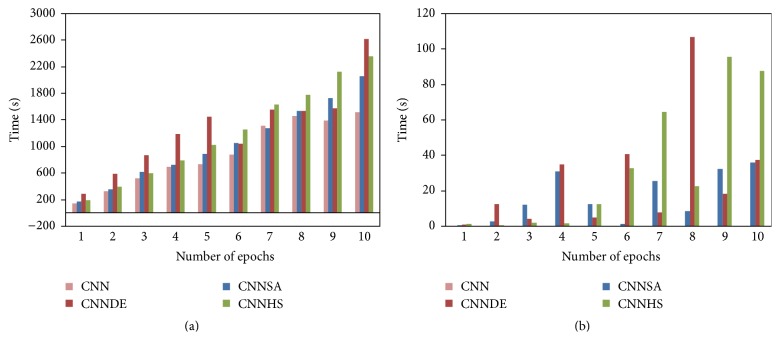
Computation time and its standard deviation (i-8c-2s-16c-2s).

All of the experiment results of the proposed methods are compared with the experiment result from the original CNN. These results for the design of i-6c-2s-12c-2s are summarized in Table 1 for accuracy, Table 2 for the computational time, and Figure 3 for error and its standard deviation as well as Figure 4 for computational time and its standard deviation. The results for the design of i-8c-2s-16c-2s are summarized in Table 3 for accuracy, Table 4 for the computational time, and Figure 5 for error and its standard deviation as well as Figure 6 for computational time and its standard deviation.

**Figure 7:**
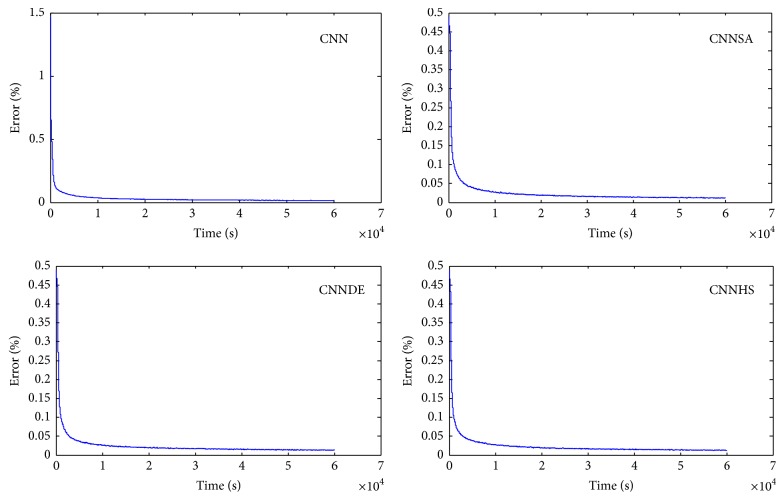
Error versus computation time for 100 epochs.

The experiments of original CNN are conducted at only one time for each epoch because the value of its accuracy will not change if the experiment is repeated with the same condition. In general, the tests conducted showed that the higher the epoch value, the better the accuracy. For example, in one epoch, compared to CNN (82.39), the accuracy increased to 5.73 for CNNSA (88.12), 3.91 for CNNDE (86.30), and 4.84 for CNNHS (87.23). While in 5 epochs, compared to CNN (93.11), the increase of accuracy is 3.18 for CNNSA (96.29), 2.04 for CNNDE (94.15), and 1.78 for CNNHS (94.89). In the case of 100 epochs, as shown in Figure 7, the increase in accuracy compared to CNN (98.65) is only 0.16 for CNNSA (98.81), 0.13 for CNNDE (98.78), and 0.09 for CNNHS (98.74).

The experiment results show that CNNSA presents the best accuracy for all epochs. Accuracy improvement of CNNSA, compared to the original CNN, varies for each epoch, with a range of values between 1.74 (9 epochs) and 5.73 (1 epoch). The computation time for the proposed method, compared to the original CNN, is in the range of 1.01 times (CNNSA, two epochs: 246/244) up to 1.70 times (CNNHS, nine epochs: 1246/856).

**Figure 8:**
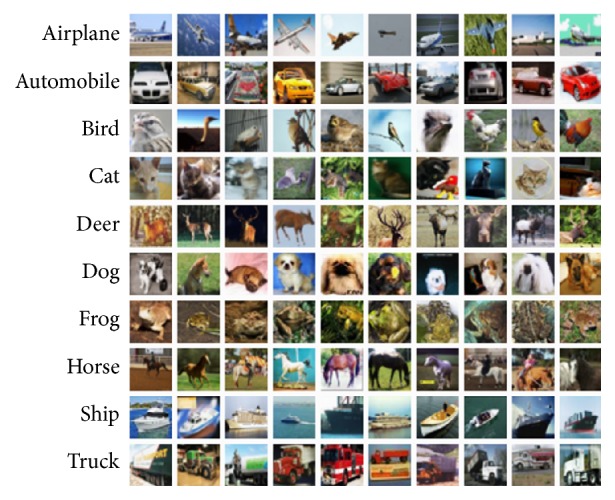
Example of some images from CIFAR dataset.

In addition, we also test our proposed method with CIFAR10 (Canadian Institute for Advanced Research) dataset. This dataset consists of 60,000 color images, in which the size of every image is 32 × 32. There are five batches for training, composed of 50,000 images, and one batch of test images consists of 10,000 images. The CIFAR10 dataset is divided into ten classes, where each class has 6,000 images. Some example images of this dataset are showed in Figure 8.

**Figure 9:**
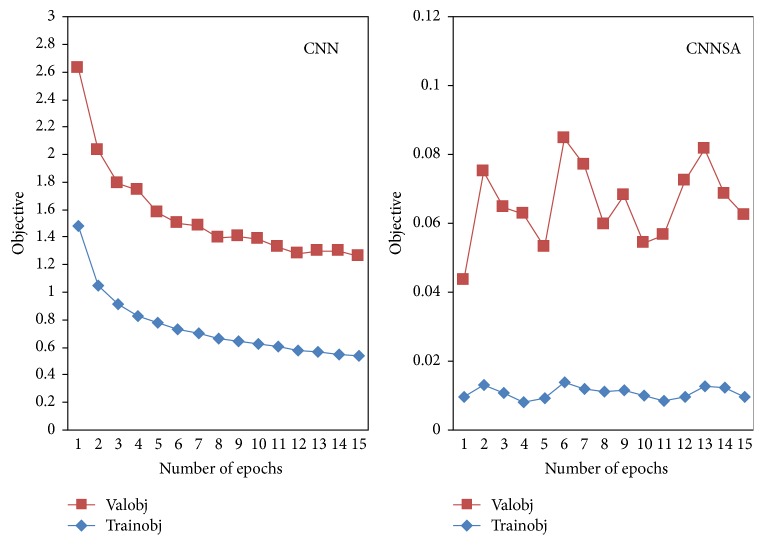
CNN versus CNNSA for objective.

**Figure 10:**
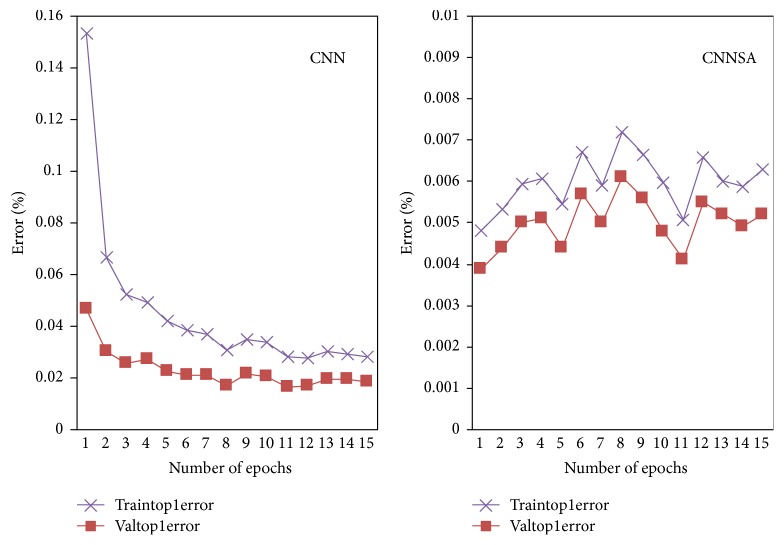
CNN versus CNNSA for top-1 error.

**Figure 11:**
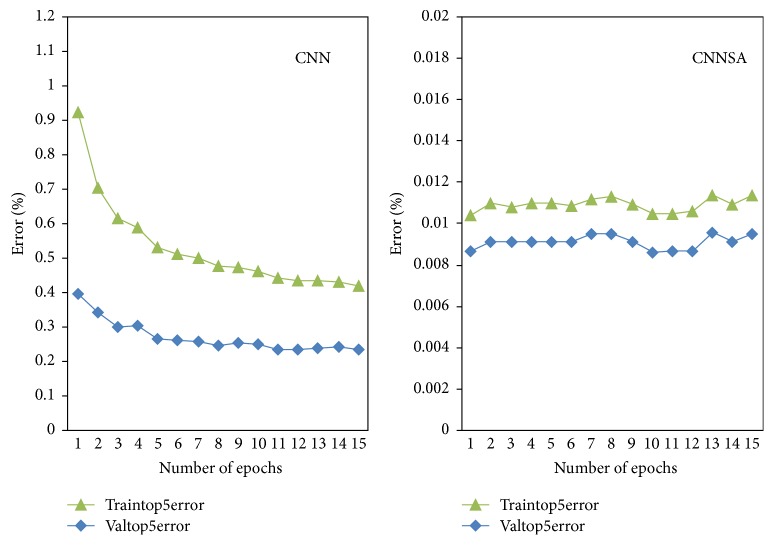
CNN versus CNNSA for top-5 error.

The experiment of CIFAR10 dataset was conducted in MATLAB-R2014a. We use the number of epochs 1 to 15 for this experiment. The original program is MatConvNet from [24]. In this paper, the program was modified with SA algorithm. The results can be seen in Figure 9 for objective, Figure 10 for top-1 error, and Figure 11 for a top-5 error, Table 5 for Comparison of CNN and CNNSA for train as well as Table 6 for Comparison of CNN and CNNSA for validation. In general, these results show that CNNSA works better than original CNN for CIFAR10 dataset.

## 6. Conclusion

This paper shows that SA, DE, and HS algorithms improve the accuracy of the CNN. Although there is an increase in computation time, error of the proposed method is smaller than the original CNN for all variations of the epoch.

It is possible to validate the performance of this proposed method on other benchmark datasets such as ORL, INRIA, Hollywood II, and ImageNet. This strategy can also be developed for other metaheuristic algorithms such as ACO, PSO, and BCO to optimize CNN.

For the future study, metaheuristic algorithms applied to the other DL methods need to be explored, such as the recurrent neural network, deep belief network, and AlexNet (a newer variant of CNN).

## Figures and Tables

**Algorithm 1:**
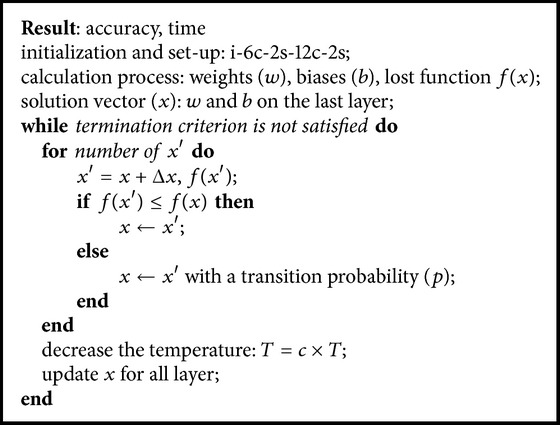
CNNSA.

**Algorithm 2:**
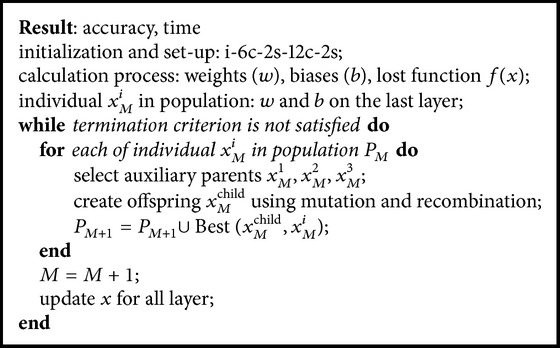
CNNDE.

**Algorithm 3:**
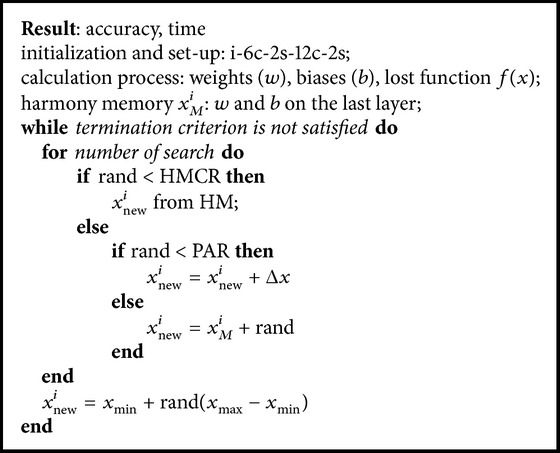
CNNHS.

**Table 1:** Accuracy and its standard deviation for design: i-2s-6c-2s-12c.

Epoch	CNN		CNNSA		CNNDE		CNNHS	
	Accuracy	Standard deviation	Accuracy	Standard deviation	Accuracy	Standard deviation	Accuracy	Standard deviation
1	82.39	n/a	88.12	0.39	86.30	0.33	87.23	0.95
2	89.06	n/a	92.77	0.43	91.33	0.19	91.20	0.33
3	91.13	n/a	94.61	0.31	93.45	0.28	93.24	0.40
4	92.33	n/a	95.57	0.16	94.63	0.44	93.77	0.12
5	93.11	n/a	96.29	0.14	95.15	0.15	94.89	0.33
6	93.67	n/a	96.61	0.18	95.67	0.20	95.17	0.43
7	94.25	n/a	96.72	0.12	96.28	0.20	95.65	0.20
8	94.77	n/a	96.99	0.11	96.59	0.11	96.08	0.24
9	95.37	n/a	97.11	0.06	96.68	0.17	96.16	0.11
10	95.45	n/a	97.37	0.14	96.86	0.10	96.98	0.06

**Table 2:** Computation time and its standard deviation for design: i-2s-6c-2s-12c.

Epoch	CNN		CNNSA		CNNDE		CNNHS	
	Time	Standard deviation	Time	Standard deviation	Time	Standard deviation	Time	Standard deviation
1	93.21	n/a	117.48	1.12	138.58	0.90	160.92	0.85
2	225.05	n/a	243.43	9.90	278.08	1.66	370.59	5.87
3	318.84	n/a	356.49	1.96	414.64	2.43	414.13	0.63
4	379.44	n/a	479.83	1.95	551.39	2.28	554.51	0.73
5	479.04	n/a	596.35	4.08	533.21	1.42	692.90	2.90
6	576.38	n/a	721.48	1.48	640.30	6.19	829.56	1.95
7	676.57	n/a	839.55	1.19	744.89	3.98	968.18	1.97
8	768.24	n/a	960.69	1.74	852.74	4.48	1105.2	1.39
9	855.85	n/a	1082.18	2.54	957.89	5.78	1245.54	4.96
10	954.54	n/a	1202.52	2.08	1373.1	1.51	1623.13	4.36

**Table 3:** Accuracy and its standard deviation for design: i-2s-8c-2s-16c.

Epoch	CNN		CNNSA		CNNDE		CNNHS	
	Accuracy	Standard deviation	Accuracy	Standard deviation	Accuracy	Standard deviation	Accuracy	Standard deviation
1	80.09	n/a	86.36	0.76	84.78	1.24	87.23	0.57
2	89.04	n/a	91.18	0.25	91.63	0.30	92.15	0.55
3	90.98	n/a	93.56	0.20	93.67	0.17	93.69	0.31
4	92.27	n/a	94.69	0.16	94.86	0.43	94.63	0.20
5	93.17	n/a	95.51	0.12	95.57	0.04	95.30	0.16
6	93.79	n/a	96.23	0.08	96.20	0.14	95.80	0.25
7	94.74	n/a	96.52	0.08	96.52	0.32	95.71	0.24
8	95.22	n/a	96.95	0.07	96.68	0.19	96.40	0.13
9	95.54	n/a	97.18	0.08	97.10	0.00	96.84	0.27
10	96.05	n/a	97.35	0.02	97.32	0.04	96.77	0.04

**Table 4:** Computation time and its standard deviation for design: i-2s-8c-2s-16c.

Epoch	CNN		CNNSA		CNNDE		CNNHS	
	Time	Standard deviation	Time	Standard deviation	Time	Standard deviation	Time	Standard deviation
1	145.02	n/a	175.08	0.64	289.54	0.78	196.10	1.35
2	323.62	n/a	353.55	2.69	586.96	12.56	395.43	0.60
3	520.16	n/a	614.71	12.10	868.82	4.02	597.391	1.83
4	692.80	n/a	718.53	31.05	1185.49	34.95	794.43	1.70
5	729.05	n/a	885.64	12.53	1451.64	4.99	1023.72	12.51
6	879.17	n/a	1051.30	1.30	1045.26	40.62	1255.93	32.54
7	1308.21	n/a	1271.03	25.55	1554.67	7.86	1627.30	64.56
8	1455.06	n/a	1533.30	8.55	15.39	106.75	1773.92	2251
9	1392.62	n/a	1726.50	32.31	1573.52	18.42	2123.32	95.76
10	1511.74	n/a	2054.40	35.85	2619.62	37.37	2354.90	87.68

**Table 5:** Comparison of CNN and CNNSA for train.

Epoch	CNN			CNNSA		
	Objective	Top-1 error	Top-5 error	Objective	Top-1 error	Top-5 error
1	1.4835700	0.10676	0.52868	0.009493	0.00092	0.00168
2	1.0443820	0.03664	0.36148	0.013218	0.00094	0.00188
3	0.9158232	0.02686	0.31518	0.010585	0.00094	0.0017
4	0.8279042	0.02176	0.28358	0.008023	0.00096	0.00188
5	0.7749367	0.01966	0.26404	0.009285	0.00106	0.00186
6	0.7314783	0.01750	0.25076	0.013674	0.00102	0.00175
7	0.6968027	0.01566	0.23968	0.117740	0.0009	0.00168
8	0.6654411	0.01398	0.22774	0.011239	0.0011	0.0018
9	0.6440073	0.01320	0.21978	0.011338	0.00106	0.0018
10	0.6213060	0.01312	0.20990	0.009957	0.00116	0.0019
11	0.6024042	0.01184	0.20716	0.008434	0.00096	0.00176
12	0.5786811	0.01090	0.19954	0.009425	0.0011	0.00192
13	0.5684009	0.01068	0.19548	0.012485	0.00082	0.0018
14	0.5486258	0.00994	0.18914	0.012108	0.00098	0.00184
15	0.5347288	0.00986	0.18446	0.009675	0.0011	0.00186

**Table 6:** Comparison of CNN and CNNSA for validation.

Epoch	CNN			CNNSA		
	Objective	Top-1 error	Top-5 error	Objective	Top-1 error	Top-5 error
1	1.148227	0.0466	0.3959	0.034091	0.0039	0.0087
2	0.985902	0.0300	0.3422	0.061806	0.0044	0.0091
3	0.873938	0.0255	0.2997	0.054007	0.0050	0.0091
4	0.908667	0.0273	0.3053	0.054711	0.0051	0.0091
5	0.799778	0.0226	0.2669	0.043632	0.0044	0.0091
6	0.772151	0.0209	0.2614	0.071143	0.0057	0.0091
7	0.784206	0.0210	0.2593	0.065040	0.0050	0.0095
8	0.732094	0.0170	0.2474	0.048466	0.0061	0.0095
9	0.761574	0.0217	0.2532	0.056708	0.0056	0.0091
10	0.763323	0.0207	0.2515	0.044423	0.0048	0.0086
11	0.720129	0.0165	0.2352	0.047963	0.0041	0.0087
12	0.700847	0.0167	0.2338	0.063033	0.0055	0.0087
13	0.729708	0.0194	0.2389	0.068989	0.0052	0.0096
14	0.747789	0.0192	0.2431	0.056425	0.0049	0.0091
15	0.723088	0.0182	0.2355	0.052753	0.0052	0.0095
